# Tools for the identification of victims of domestic abuse and modern slavery in remote services: A systematic review

**DOI:** 10.1177/13558196241257864

**Published:** 2024-06-07

**Authors:** Bella Tomsett, Johanna Álvarez-Rodríguez, Nigel Sherriff, Natalie Edelman, Anne Gatuguta

**Affiliations:** 1 School of Sport and Health Sciences, 1947 University of Brighton, Brighton, UK; 2 Centre for Health Services Studies, University of Kent, Canterbury, UK; 3 Independent Consultant, Trauma-informed Research, Support & Training (TRuST), Lewes, UK; 4 Department of Global Health and Infection, Brighton and Sussex Medical School, University of Sussex, Brighton, UK

**Keywords:** domestic abuse, modern slavery, remote services

## Abstract

**Objective:**

To explore the technology-based tools available for supporting the identification of victims of domestic abuse and modern slavery in remote services and consider the benefits and challenges posed by the existing tools.

**Methods:**

We searched six academic databases. Studies were considered for inclusion if they were published in English between 2000 and 2023. The QuADS quality appraisal tool was used to assess the methodological quality of included studies. A narrative synthesis was conducted using the convergent integrated approach.

**Results:**

Twenty-four studies were included, of which two were professional guidelines; each reported on a distinct technology-based tool for remote services. All tools related to domestic abuse and 21 focused on screening for intimate partner violence among young and mid-life women (18–65) in high-income countries. The review did not identify tools that support the identification of victims of modern slavery. We identified eight common themes of tool strengths, highlighting that the remote approach to screening was practical, acceptable to victims, and, in some circumstances, elicited better outcomes than face-to-face approaches. Five themes pointed to tool challenges, such as concerns around privacy and safety, and the inability of computerised tools to provide empathy and emotional support.

**Conclusions:**

Available technology-based tools may support the identification of victims of domestic abuse by health and social care practitioners in remote services. However, it is important to be mindful of the limitations of such tools and the effects individuals’ screening preferences can have on outcomes. Future research should focus on developing tools to support the identification of victims of modern slavery, as well as empirically validating tools for screening during remote consultations.

## Background

Domestic abuse and modern slavery are violent phenomena that impact significantly on the physical and mental wellbeing of the individuals subjected to them.^1–3^ Domestic abuse can be defined broadly as any act or pattern of behaviour intended to cause physical, emotional, psychological and/or financial harm to a person who has a familial connection, or who is/has been the intimate partner of the perpetrator.^4^ Modern slavery refers to “situations of exploitation that a person cannot refuse or cannot leave because of threats, violence, deception, abuse of power or other forms of coercion.”^5, p.2^ This includes experiences such as forced labour, domestic servitude, and human trafficking, with a focus on the exploitation of any person, irrespective of age or gender.

Although domestic abuse and modern slavery are distinct phenomena, there are notable overlaps, in particular around the exertion of power and control over victims. Examples of domestic abuse and modern slavery intersections are sexual and reproductive coercion (whether on an interpersonal or commercial level), physical confinement and/or control of finances to prevent a person from leaving, or the use of threats and/or physical violence to keep an individual trapped.^6^ However, there is a lack of unified global definitions for domestic abuse and modern slavery and this lack significant shapes how the issues are framed and addressed across various geographical contexts, and inevitably, the nature and scope of systems and tools to support victims.

Domestic abuse and modern slavery are often invisible in society, as experiences of coercive control, fear, shame and stigma can lead to a reluctance to disclose experiences of abuse.^4,7^ Although it can be difficult to quantify domestic abuse and modern slavery, the World Health Organization (WHO) estimated that one-third of all women globally have experienced physical and/or sexual violence within a relationship.^4^ Much less is known about men’s experiences of domestic abuse. Prevalence rates of physical violence against men range from 3.4% to 20.3%^8^; for the UK it has been estimated that one in three victims of domestic abuse are male.^9^ It has further been estimated that in 2021, 50 million individuals were living in modern slavery globally, a 10 million rise from 2016.^5^

There is a need for health and care services to regularly screen for potential victims of abuse and so improve identification rates and provide appropriate support, with guidance available for health care practitioners to routinely enquire about domestic abuse.^1^ Screening is usually conducted face-to-face, but following the COVID-19 pandemic, there is increasing interest in the potential of remote technology to screen for victims of domestic abuse and modern slavery.^10–12^ However, such interventions require a strong evidence base, supported by tested and verified tools, and they should be suitable for the populations they serve.

In this paper we report a systematic review of the available literature about tools which were developed to support the identification of victims of domestic abuse and modern slavery remotely, or which are used for this purpose by providers of remote health and social care. The review specifically focused on (i) the types of tools that exist to support the remote identification of victims of domestic abuse and/or modern slavery; (ii) the evidence of effectiveness of such tools to identify victims of domestic abuse and/or modern slavery; and (iii) the limitations of existing tools.

## Methods

This mixed methods review was conducted and reported in accordance with PRISMA guidance^13^ and registered on PROSPERO (CRD42023377767). The review was conducted between 01/01/23 and 31/08/23. We did not undertake extensive stakeholder consultation prior to the review. However, informal conversations with health and social care practitioners, and with third sector organisations working with victims of domestic abuse and/or modern slavery, highlighted an interest in the topic and a need to better understand this area of practice.

### Search strategy

Four key concepts were identified using PICo criteria - **(P)**opulations: (i) people experiencing domestic abuse and/or (ii) people experiencing modern slavery; phenomenon of **(I)**nterest: (iii) victim identification; **(Co)**ntext: (iv) technology-mediated services Each concept was expanded with relevant keywords through exploratory searches in SCOPUS and Cochrane Library The search terms were combined using Boolean operators, and the ‘explode’ function was also used to identify key concepts/MeSH terms, where available (see also Online Supplement Table S1).

### Eligibility criteria

Studies were included if they: were available in English; reported data on individuals aged 16 and over; and reported on tools, systems or processes that support the identification of victims of domestic abuse, modern slavery, or both, in remote service provision. Exclusion criteria were: publication before 2000; reported data related only to children under 16; reported tools were used during face-to-face contact only; reported tools were not used to screen for victims (e.g. psychosocial and therapeutic intervention tools, violence prevention tools, educational tools); or reported tools were used to mine existing data (e.g. from electronic patient records), unless additionally linked to the development of a screening tool.

This review focused on tools designed to use with adults and older teenagers. Tools aimed at identifying victims of child abuse and exploitation were excluded as they are likely to have different considerations in terms of language (such as simplifying or adapting this to be understood by young children), consent, and legal responsibilities, which would not be relevant to an older population. However, we still intended to consider tools that incorporated screening of younger children as well as those aged 16 years and over as they might have offered valuable contributions and insights to this study. Nevertheless, no such studies were identified in practice.

We only considered studies in English because of time and resource limitations. We considered quantitative, qualitative and mixed-methods peer-reviewed studies to allow for a comprehensive exploration of this under-researched topic. We did not include systematic and scoping reviews, but searched the reference lists of any applicable reviews to extract relevant studies. We did not impose geographical restrictions to ensure a broad overview of the available evidence from various settings and populations.

### Study selection

We searched six databases between 31 January and 13 February 2023: SCOPUS, MEDLINE, PsycInfo, CINAHL, and IBSS. The Cochrane Library database was also searched to identify any relevant systematic reviews. We further hand-searched included studies’ lists of references, and we used the ‘Connected Papers’ website^14^ to identify further relevant sources. All records were exported to the Zotero reference manager (v.6.0.30), and duplicates removed. Two researchers screened all records independently for title, abstract, and full-text. Any disagreements were resolved through discussion, with a third researcher consulted as needed.

### Data extraction and quality assessment

BT and JA independently extracted data on study aims, design, participants, types of technology-based tools, tool strengths and limitations from a 10% sample of the included studies using a table that was developed for this study. Due to high level of concordance between the researchers, the remaining extraction was completed by BT alone. Studies were quality assessed using the Quality Assessment with Diverse Studies (QuADS) tool, which provides a single set of questions to query the methodological quality of quantitative, qualitative and mixed-methods studies, and was found to have good content validity and inter-rater reliability.^15^ Two researchers independently assessed a 10% sample, and a third researcher reviewed 30% of the completed quality assessment. We did not exclude any studies based on the quality assessment, but study quality was taken into consideration in data analysis and we discuss the impact of study quality on the results.

### Data analysis

Data analysis was conducted by BT using the convergent integrated approach, according to which all qualitative data and quantitative data should be extracted from relevant studies, and data should be converted into a ‘mutually compatible format’.^16^ Following this approach, we converted all quantitative data into descriptive text. We first extracted data from the results and discussions of all included studies and coded these deductively in relation to the strengths and limitations of technology-based tools in screening for abuse. Data were then analysed separately under each of the two subheadings. Through iterative examination and thematic coding, common themes were drawn out under each subheading.

## Results

**Figure 1.**
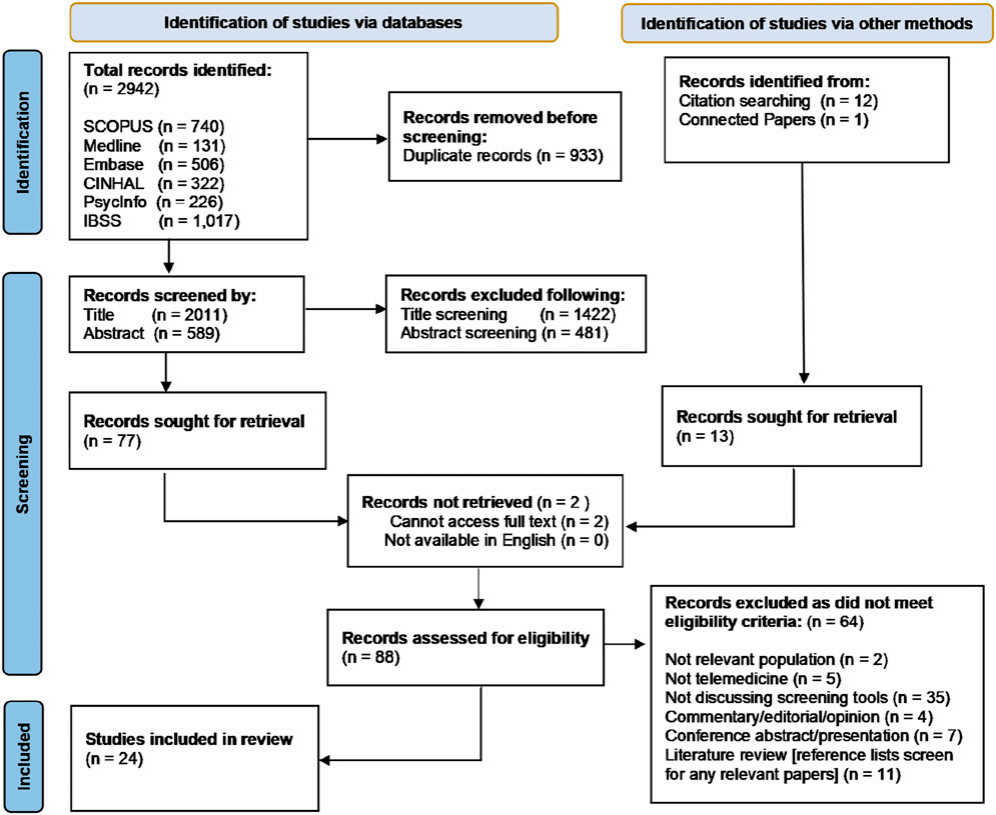
Adapted PRISMA 2020 flowchart of study selection for inclusion in the systematic review.

Our initial searches identified a total of 2942 records, of which 88 records were retrieved for full text screening against the inclusion and exclusion criteria following de-duplication and title and abstract screening. Twenty-four studies were retained for inclusion in the systematic review (Figure 1).

Table 1 provides a summary of included studies’ characteristics (please see Online Supplement Table S2 for further detail). Of the 24 included studies, four studies reported on tool development,^12,17–19^ 18 on the evaluation of tools, and two provided professional guidance for remote screening.^10,11^ All studies were conducted in high-income countries, mainly the USA (*n* = 20), with only one study set in a non-English speaking country (Portugal).^20^ The majority of studies was published after 2010. Twenty-one studies discussed intimate partner violence, two studies explored domestic violence more broadly, and one focused on elder mistreatment. We did not identify studies focusing on modern slavery. Of the 21 studies that reported on the study setting, all but one^21^ were situated within health care.

**Table 1.** Summary of studies’ characteristics and included tools.

	*N*	%
Year published		
2002	1	4.2
2006	3	12.5
2007	1	4.2
2009	1	4.2
2011	2	8.3
2012	2	8.3
2014	2	8.3
2015	3	12.5
2016	1	4.2
2020	1	4.2
2021	5	20.8
2022	2	8.3
Study type		
Randomised trial	9	37.5
Quantitative (not RT*)	6	25
Mixed methods	4	16.7
Qualitative	3	12.5
Practice guidance (non-empirical)	2	8.3
Study location		
Canada	3	12.5
Multi (Canada, USA, UK)	1	4.2
Portugal	1	4.2
USA	19	79.1
Abuse type		
Domestic violence	2	8.3
Elder mistreatment	1	4.2
IPV*	21	87.5
Service type (Data available from *N* = 21)		
General ED*	6	28.6
Women's health	6	28.6
Paediatric ED*	3	14.3
Primary care	3	14.3
Non-specific health care services	3	14.3
WIC* centre	1	4.8
Probation services	1	4.8
Professionals involved in screen (Data available from *N* = 16)		
Doctor	9	56.3
Nurse	10	62.5
Medical assistant	2	12.5
Social worker	3	18.8
Pre/peri-natal health care provider	3	18.8
Probation case manager	1	6.3
Unspecified clinician	2	12.5
Type of tool		
Computer-based questionnaire	9	37.5
Web-based app/programme	6	25
Guidance for remote consult	3	12.5
EPR* data mining	2	8.3
A-CASI*	2	8.3
Telephone-based questionnaire	1	4.2
Audiotape questionnaire	1	4.2
Tool purpose		
Screening	22	91.7
Information	11	45.8
Education	6	25
Pre-screen	2	8.3
Conversation starter	2	8.3
Referral	1	4.2
Intervention	1	4.2
Motivation	1	4.2
Tools with more than 1 function	15	62.5
Mode of remote application		
Tablet/mobile	9	37.5
Computer/laptop	9	37.5
Remote consultation (phone/video)	4	16.7
Headset	3	12.5
Computer algorithm	2	8.3
Touch-screen kiosk	1	4.2
Existing abuse screening questionnaires used/adapted		
Partner violence screen	7	29.1
Abuse assessment screen	5	20.8
FVPF* resource manual for HCP*	2	8.3
Revised conflict tactics scales–Short form	2	8.3
Danger assessment	2	8.3
Woman abuse screening tool	2	8.3
Women’s experience with battering scale	2	8.3
Elder abuse suspicion index	1	4.2
Composite abuse scale	1	4.2
Hurt, insult, threaten, and scream	1	4.2
Feldhaus partner violence screen	1	4.2
CDC* behavioral risk factor surveillance system	1	4.2
Medical advocacy screening questionnaire	1	4.2
Psychological maltreatment against women inventory	1	4.2
Total instruments	14	

*Abbreviations: RT - randomised trial**; IPV -** intimate partner violence**; ED -** emergency department**; WIC -** women, infants and children**; EPR -** electronic patient records**; A-CASI -** audio-computer assisted self-interview**; FVPF -** family violence prevention fund**; HCP -** health care providers**; CDC -** centers for disease control and prevention

### Participant demographics

Table 2 presents demographic information about study participants. Participant characteristics were not systematically reported. Of the 21 studies that did so, 15 recruited only women and six included men (an average 21% of the total sample). We did not identify any studies that reported on transgender or gender-nonconforming individuals and only one explicitly considered non-heterosexual relationships.^21^ Fifteen studies provided detailed information on participants’ age, which ranged from 16–98 years (mean = 35.4 years). In the studies providing information about participants’ ethnicity (*N* = 13; 5882 participants), the majority were Black/African-American (57.6%), followed by White (28.7%) and Latin/Hispanic (6.8%). Five studies included professionals as participants within their design (assessing professionals’ use of the tool), of which the majority were doctors. One study that explored elder mistreatment considered family caregivers within its participant sample.^22^

**Table 2.** Summary of participant characteristics.

Total participants (*N* = 21)	437,223
Sample range	17 - 405,303
Professionals as participants (N = 5)	172
Sample range	5 - 80
Doctors	128
Nurse practitioners	8
Social workers	3
Unspecified clinicians	2
Software designers	2
Midwives/perinatal professionals	29
Age (*N* = 15)	
Mean age	35.4
Age range	16 - 98
Gender (*N* = 21)	
Women only	15
Women and men	6
Where men included, avg. proportion of sample	21%

	*N*	%
Ethnicity (*N* = 13; 5882 people)		
Hispanic/Latin	398	6.80%
Black/African American	3389	57.60%
White	1690	28.70%
Asian	18	>1%
Native American	1	>1%
Mixed/other	310	5.30%
Not known	88	1.50%
Marital status (*N* = 12; 20,493 people)		
Married/in relationship	8947	43.60%
Single	7367	36.00%
Divorced/separated/widowed	2420	11.80%
Not known	1772	8.60%

### Identified tools

All identified tools aimed at the identification of victims of domestic abuse; the majority restricted their applicability to intimate partner violence (Table 1). Most tools were designed to enable screening for domestic abuse either by professionals or by victims themselves. Fifteen of the 24 studies presented a tool which served more than one purpose (e.g. screening and information). Fourteen tools were designed for use with women, ten for women and men, and none for men alone.

The majority of tools were designed to be used with computers, laptops, tablets or mobile phones for remote delivery. Four studies discussed remote consultations by phone or video and three incorporated audio into the process for added privacy and inclusion of participants with low literacy. Fourteen tools designed for in-person screening for various types of abuse, mostly intimate partner violence, were utilised or adapted within the reviewed studies. Of those, the most common were Partner Violence Screen^23^ and Abuse Assessment Screen,^24^ selected for their tested validity, reliability and brevity.

### Quality assessment

The diversity of study designs and reporting formats made the systematic assessment of methodological quality challenging. Overall, reviewed studies were of variable methodological quality, with QuADS scores ranging from 14 to 36 of possible 39 points (see Online Supplement Table S3).

Most studies scored highly in the domains of research aims (Q2) and the setting and target population (Q3), indicating that they made the study purpose clear, and the setting and population of the study explicit. Studies were consistently weak on theoretical and conceptual underpinnings of the research (Q1) and stakeholder involvement (Q12), and most studies made no reference to either. Although the analytical method chosen appeared to be largely appropriate to address the research aims (Q10), many studies did not provide explicit justification for their choice.

Reviewed randomised trials tended to not blind participants and/or professionals to the interventions. Although full blinding may be difficult to achieve when testing screening tools, there is a risk of performance bias which could affect the validity of the results, and few studies reflected on this limitation. Several of the studies reported to measure acceptability, but few incorporated qualitative methods into their design to fully explore this claim.

As noted above, we did not exclude any studies on the basis of quality assessment, and the QuADS guidance does not offer a cut-off score for quality. Instead, we interpreted the evidence with the methodological limitations in mind. We were unable to assess the methodological quality of two studies,^10,11^ as these provided practice guidance based on literature only. We still included these as they highlighted an important gap in the empirical literature.

### Strengths and limitations of identified tools

We identified eight themes on how technology-based tools in remote services can be effective in supporting the identification of victims of domestic abuse and present opportunities for improved usability, accessibility, and acceptability (‘strengths’). Limitations included five themes, which related to either the identification of victims or the implementation of the remote approaches themselves (Table 3). We report on the two headings in turn.

**Table 3.** Mapping of themes against included studies.

		Abujarad, F., et al. (2021)	Ahmad, F., et al. (2009)	Bacchus, L.J., et al. (2016)	Bair-Merritt, M.H., et al. (2006)	Bhargava, R., et al. (2011)	Chang, J., et al. (2012)	Choo, E., et al. (2015)	Fincher, D., et al. (2015) x	Fraga, S., et al. (2014)	Gilbert, L., et al. (2015)	Gottlieb, L., et al. (2014)	Jack, S.M., et al. (2021)	Klevens, J., et al. (2012)	Krishnamurti, T., et al. (2021)	MacMillan, H. L., et al. (2006)	O'Campo, P., et al. (2021)	Ragavan, M.I., et al. (2020)	Rhodes, K.V., et al. (2002)	Rhodes, K.V., et al. (2006)	Scribano, P.V., et al. (2011)	Simon, M.A. (2021)	Spence, E.E., et al. (2022)	Tabaie, A., et al. (2022)	Trautman, D.E., et al. (2007)
Effectiveness & Advantages	Remote screening could be a practical way to increase screening	x	x								x	x		x	x					x	x		x		
	Computers may be more effective in facilitating disclosure on sensitive issues		x	x	x							x		x						x					**x**
	Some individuals prefer computerised screening over face-to-face			x	x		x	x								x		x							
	Remote screening can be used to complement face-to-face screening					x							x		x		x	x	x	x		x		x	**x**
	Screening for DA could be done alongside screening for other psychosocial issues		x			x		x				x						x	x				x		
	Importance of the professional in remote screening			x			x		x				x	x				x	x		x	x			**x**
	Remote screening associated with higher rates of referral for support										x									x					**x**
	Language used in screening tool is important to engagement and outcomes							x									x								
Barriers & challenges	Increase in use of remote tools as a result of the COVID-19 pandemic												x		x		x	x				x			
	Need to consider safety/privacy when screening	x	x	x	x			x					x	x				x			x	x	x		
	Need to be mindful of the role of the perpetrator			x	x								x					x				x	x		
	Computerised tools unable to provide empathy, emotional support and meaningful feedback			x			x	x																	
	Need for staff training			x									x								x	x			

### Strengths of remote services to identify victims of domestic abuse

Of 18 studies evaluating tool effectiveness, 11 found that remote approaches were comparable or better than face-to-face approaches in facilitating disclosure and improving the identification of victims of domestic abuse;^21,22,25–33^; some individuals expressed a preference for computerised screening over in-person approaches.^10,28,34–37^

#### Fully-computerised screening enhances reporting of sensitive issues

Seven studies^25–28,30,31,34^ reported that fully digital screening (where questions are asked by a computer rather than a person) may better enable disclosure of more stigmatised issues, such as partner violence and substance (mis)use. Participants in one study^36^ described feeling shame in relation to the abuse they were experiencing, citing their perception of computers as non-judgemental as a contributing factor to their decision to disclose. Lack of judgement was also identified as a facilitator by Chang et al.^37^ Other studies noted that computerised screening enhanced participants’ sense of privacy, anonymity and safety,^22,33^ reduced risk of re-traumatisation,^28^ and in some cases, improved accessibility to screening and support.^10^

#### Screening modality can improve disclosure of abuse

In most cases, remote screening elicited higher rates of disclosure, with individuals reporting a preference for such approaches. Only two reviewed studies found that participants were most likely to disclose abuse during face-to-face conversations; of these, one focused on low-income African-American women,^38^ and the other was conducted in Portugal.^20^ Fraga et al. speculated that a preference of Portuguese women could be due to cultural norms, such as favouring a personal approach when discussing negative experiences. Rhodes et al.^31^ found that people in urban settings disclosed higher rates of intimate partner violence than those living in suburban settings; they were also more likely to report satisfaction with the service if domestic abuse was discussed. These findings point to some variation in preferences and needs around domestic abuse screening.

Several studies noted that computerised screening for domestic abuse could be undertaken alongside screening for other psychosocial issues, such as drug use or mental health challenges,^19,25,27,29,33,36,39^ and so potentially capture several issues in a single intervention. Gottlieb et al.^25^ conducted a randomised trial, screening for 23 psychosocial areas of need, including domestic abuse. They found that 96.8% of participants disclosed concern in relation to more than one area of need, and reported difficulties in relation to ten of the 23 areas, on average. Here, computerised screening was found to be particularly effective in facilitating disclosure from low-income families, and those experiencing domestic abuse.

#### Remote screening for abuse has practical value

Nine studies concluded that even where remote services elicit similar results when compared with face-to-face screening, they could provide a practical way to increase screening.^21,22,25–27,29,31–33^ This is because remote interventions can be relatively low-cost and simple to implement, especially in settings where digital infrastructures are already in place, while reducing the demand on professionals’ time. Three studies^19,32,39^ proposed that remote tools could be one way to improve on the low rates of screening and documentation of domestic abuse in health care settings, increasing adherence to local policies on routine enquiry, and the likelihood of support for victims.

Other studies highlighted the potential of remote tools to complement face-to-face screening.^10–12,17–19,30–32,39^ For example, an algorithm applied to electronic patient records could alert clinicians to the need to screen particular individuals for domestic abuse due to identified risk.^18,19^ Tools for screening during remote servicess could also be used as a way to triage individuals to in-person appointments on the basis of identified risk or need.^10–12^

#### Support from professionals improves remote services implementation

The importance of professionals in remote services was highlighted in almost half of the included studies.^10–12,26,28,30,33,37–39^ Professionals’ involvement, knowledge and skills were identified as instrumental to the successful implementation of all types of remote screening approaches. Scribano et al.^33^ found that uptake of a computerised self-screen increased substantially once nurse champions were introduced in the emergency department, encouraging and supporting individuals to screen. In another study, clinician participants highlighted the importance of the relationship they build with their patients in order to know when to offer the computerised screen.^28^ In the same study, some patient participants reported that they were more likely to share a positive screen for intimate partner violence if they trusted their health care provider, as they would feel able to seek support and reassurance from them. Rhodes et al.^39^ noted, however, that although computerised screening might enhance disclosure of abuse, professionals would need to undertake risk assessments and safety planning if the process is to extend beyond identification, to the provision of meaningful support to victims.

#### Attention to language can enhance engagement and outcomes

Two studies highlighted the importance of language to encourage engagement with technology-based tools and facilitate successful screening. O’Campo et al.^17,p.12^ incorporated “affirmative and strength-based language and phrases”, as well as “language that simultaneously prioritized trusting users’ own instincts about maintaining safe behaviors while also encouraging safety planning action” (e.g. “you know your situation best”) into the design of their screening tool. This empowered the women in the study to make autonomous decisions about the disclosure of abuse and support planning.

Choo et al.^36^ further noted that the terminology used to describe the tools themselves could have an impact on individuals’ willingness to engage. Their study found that some participants differentiated between computers (which they perceived to be outside the scope of their abilities) to mobile phones (which they used to perform computer-based functions on a regular basis). For these participants, enabling screening via a mobile phone was more likely to support engagement with the process. Both studies highlighted the value of incorporating intended end-users in the design and development of remote approaches for the identification of victims of domestic abuse.

### Limitations of remote services to identify victims of domestic abuse

#### Privacy and safety

Almost half the included studies highlighted the need to consider the safety and/or privacy of the individuals being screened using remote tools.^10,11,11,12,22,26–29,33,34,36^ For example, in their study of computer-assisted screening to improve the detection of women at risk for intimate partner violence in a family practice setting, Ahmad et al.^27^ reported that participants raised concerns about the ability of the clinical setting and the professionals within it to offer adequate privacy to facilitate disclosure safely regardless of the mode of screening. Similarly, in one trial comparing women's acceptability of two different intimate partner violence screening methods in a paediatric emergency department, study participants were reluctant to discuss abuse if they had older children with them.^34^ There was concern that disclosure during a computerised screen could be brought up during the face-to-face appointment.

Privacy and safety concerns were seen to be of particular importance with regard to the perpetrator of violence and abuse. Adaptations such as adding reflective screens for digital screening tools using a computer or a mobile device, were identified to increase privacy and safety, as these made it difficult for anyone other than the person using the device to see the content.^29^ Likewise, the incorporation of safety mechanisms into the screening programme allows the individual to disguise the content quickly and safely.^28^ In addition, the use of audio and headphones was proposed as a possible safety measure for added privacy.^26,34^ However, some participants also emphasised that abusers may monitor or control access to digital devices, making some types of computerised screening not feasible.^36^ The role of the perpetrator needs to be considered carefully when discussing abuse during remote consultation, as it can be difficult to establish whether the person is alone and able to talk safely.

#### Lack of screening tools

As noted above, the COVID-19 pandemic accelerated the use of remote services and this was recognised in a number of the reviewed studies.^10–12,17,32^ At the same time, studies reported on the limited tools available to respond to domestic abuse appropriately using remote services and this was seen to be particularly relevant for screening by clinicians during remote consultation, for which no validated or tested tools were identified. It was noted that suitable tools needed to be developed to ensure victims are not missed in remote consultations, and such tools should be tested to ensure they achieve the desired objectives in real-world encounters. Spence et al.^29^ and Jack et al.^12^ reported on the development of new tools to support screening using remote consultation but these continue to require testing in practice.

#### Lack of confidence and trust in remote tools

Participants in three studies^28,36,37^ expressed uncertainty about the ability of a computerised tool to respond to disclosures of abuse in a sensitive, supportive and meaningful way. Linking with the above mentioned importance of the professionals in the screening and disclosure process, participants also suggested that digital devices could supplement, but not replace, human interaction when disclosing abuse. They expressed a need for validation, connection and empathy, all of which were seen to be outside the capability of a computerised tool.

In addition, although several studies reported that remote screening was associated with increased referrals for support for individuals identified as experiencing abuse compared to standard care,^21,30,31^ such referrals did not guarantee individuals engaging with the support on offer. For example, Klevens et al.^26^ conducted a study in a facility with an on-site intimate partner violence advocacy service to which individuals disclosing abuse were referred for support. However, although computerised screening improved disclosure and referral rates, none of the individuals disclosing abuse accessed the advocacy services in the three months following referral. Furthermore, participants who were given a list of resources by the health care provider in person reported to be more likely to use this to contact services than participants who were provided the same list by the computer.

#### Professionals’ knowledge and attitudes

While the importance of professionals was highlighted as noted above, a number of studies stressed that staff are appropriately trained for remote consultation to be effective in the identification of victims of abuse.^11,12,28,33^ For instance, Scribano et al.^33^ identified that participants’ self-screening rates improved significantly when staff within the study setting understood the value of screening. Bacchus et al.^28^ explored perinatal home visitors’ and women’s experiences of screening for intimate partner violence using mHealth technology and stressed the importance of staff training to enable them to integrate this technology into their daily practice. Likewise, Jack et al.^12^ and Simon^11^ viewed staff education and training as essential to allow professionals who deliver remote consultations develop the skills required to recognise and respond to the needs of individuals experiencing abuse.

## Discussion

To the best of the authors’ knowledge, this is the first systematic review exploring tools for the identification of victims of domestic abuse and/or modern slavery remotely. Our findings point to the potential for a combined screening process to support the identification of victims of domestic abuse and modern slavery, which may improve screening rates by time-poor practitioners in various settings. However, only a small number of reviewed studies reported on the specific types of abuse identified in detail, and as we did not identify studies that discussed modern slavery specifically. We were unable to explore this issue further. Existing reviews have considered technology-based interventions for victims of domestic abuse,^40,41^ but they tended to focus on intimate partner violence and available support in the form of information, education and therapeutic input, but without specific consideration of the identification of victims.

Our review points to some important gaps in the available literature. Thus, we were unable to identify tools relevant to modern slavery and this highlights the need for further tool development to ensure practitioners are equipped to screen for modern slavery so that victims can be offered timely and appropriate support. Moreover, there was a notable paucity of empirical research concerning the use of technology to identify and support victims of abuse during remote consultations. Of the three studies that specifically considered tools for use during technology-mediated appointments, two were sets of professional guidelines published during the COVID-19 pandemic rather than empirical studies. We included these as they highlight the lack of more evidence-based literature regarding remote screening. This, of course, does not mean that local tools and processes were not developed in practice to address the specific needs of screening for abuse using remote technology. For example, Cortis et al.,^42^ conducting a survey of practitioners working in domestic and family violence services in Australia, found that 40% of respondents had adapted existing tools in response to the new circumstances created by the COVID-19 pandemic. However, over half of respondents said that they had used the same tools without any modifications.

Furthermore, our review exposes a need to understand better the reasons for disclosure preferences of certain groups to ensure that opportunities to provide appropriate support to people experiencing abuse are maximised, and that services can tailor their interventions and target their resources to benefit the public they serve. Such understanding might be best achieved through extensive qualitative examination of the needs, motivations, and preferences of victims of abuse, of which this review found limited evidence. Moreover, the need to adequately respond to the complex needs of diverse populations supports the idea that telemedicine and face-to-face interventions for victims of abuse should be complementary rather than exclusionary, to maximise the likelihood of victim identification.

Our review further revealed that relevant studies were all conducted in high-income countries, largely the USA (80%). Yet, remote technology is used extensively in low- and middle-income countries (LMIC)^43^ and this apparent lack in the development, evaluation and reporting on evidence-based tools in LMIC settings can result in further increasing the global gap between populations which have access to appropriate services and those that do not. Geographical location can also affect the socio-demographic makeup of study populations. For example, reviewed studies reported recruiting a majority of White, Black/African-American and Latin/Hispanic participants, which correspond to the major ethnic groups in the USA.^44^ Conversely, fewer than 1% of the participants in reviewed studies were of East-Asian descent, despite Han-Chinese people representing 19% of the global population^45^ and Asia being the most populated continent, and, importantly, the high and increasing prevalence of domestic violence in many Asian countries and among migrant Asian communities.^46^ However, in the absence of research incorporating the relevant populations, any tools developed would not necessarily be appropriate for use with members of those communities.

Other notable omissions in the socio-demographic distribution of the participants in the included studies related to age, sexual orientation and gender diversity. Few studies reported recruiting participants aged 16-18 or over 65 years, and the gap was particularly notable with regard to older people’s needs. It is unclear whether this omission is due to prejudice-based bias in recruitment, with older people being viewed as less likely to experience domestic abuse or be perceived as less comfortable with using technology. However, Abujarad et al.^22^ found that most older people reported having access to and feeling comfortable using computers and mobile devices, as well as finding these acceptable tools for abuse screening. Global figures estimate that one in six older people experience some type of abuse,^47^ including domestic abuse and modern slavery. This suggests that it will be important to ensure that suitable tools are developed to enable the identification of older people experiencing such abuse, reflecting their particular circumstances and needs.

Similarly, research points to members of the LGBTQ + community experiencing a higher prevalence of domestic abuse, with additional modes of victimisation, and barriers to disclosure related specifically to the victims’ sexual orientation and/or gender.^48,49^ However, only one reviewed study reported on participants’ sexual orientation and we did not identify any work that reported on the participants’ gender identity. Furthermore, the disproportionate focus on intimate partner violence in reviewed studies may have also precluded the identification of other forms of domestic abuse, such as parental abuse, abuse by children towards parents, and sibling abuse. This is significant in the context of transgender individuals who are at high risk of emotional, physical and sexual abuse from family members.^49^ It has been suggested that the COVID-19 pandemic significantly exacerbated domestic abuse for LGBTQ + individuals who were experiencing abuse prior to the start of the pandemic,^50^ and it will be crucial to ensure inclusion of these populations to improve the identification of victims.

Reviewed studies were of variable methodological quality, although the majority of those that calculated rates of domestic disclosed through remote technology, found these to be comparable to the documented prevalence in similar populations and settings. However, it is important to note that the lack of systematic reporting on socio-demographic information made it difficult to consider the differential effects of personal attributes in relation to screening for abuse remotely. Future studies should incorporate such analysis to develop tools that are accessible and acceptable to all individuals who may require them.

Finally, although many of the included studies incorporated tool acceptability into their outcome measures, few included qualitative analysis. Such analysis is however needed to understand fully the impact of the intervention on the target population and ensure that it is meeting their needs; such understanding may increase the likelihood of intervention uptake beyond the study period.^51^

This systematic review is situated within a larger research study, which aims to develop a tool to support the identification of victims of domestic abuse and/or modern slavery in remote consultations. The findings of this review will inform planned research addressing some of the identified gaps, aiming to improve the accessibility and acceptability of technology-mediated screening to victims and professionals.

### Study limitations

This systematic review has a number of limitations. Firstly, data extraction, analysis and synthesis was mostly undertaken by a single researcher. However, the use of additional reviewers during the screening, data extraction and quality assessment stages was a mitigating factor, and the high level of concordance between the researchers was encouraging. Second, a particular challenge of this review was converting quantitative data into qualitative narratives, introducing an additional layer of data interpretation prior to the analysis. However, because of the comparatively small number of included studies, and their heterogeneity, the convergent integrated approach was considered to be appropriate to present findings which are meaningful and useful. Third, we restricted studies to those written in English language because of budget restrictions that would not have allowed for translation. This may have resulted in relevant literature being missed, especially from non-English speaking countries. Finally, we aimed to explore tools for the identification of victims of domestic abuse and modern slavery but were unable to identify studies that considered modern slavery. This highlights the lack of relevant empirical literature, a gap that requires addressing given the ongoing rise in modern slavery and increased use of remote technology in health and social care settings. We did not conduct searches of the grey literature and this may have resulted in missing reports documenting relevant research and tools.

## Conclusion

This review contributes to the literature on tools developed for the identification of victims of abuse using remote technology. It finds that remote technology can be an acceptable and practical way to screen for victims of abuse by service users and professionals. It also highlights that when such tools supplement (rather than replace) face-to-face approaches, this may result in overall higher rates of screening and victim identification. In addition, we identify important gaps in the published literature, mainly concerning the identification of victims of modern slavery, a lack of empirical data about tools that support victim identification during remote consultations, and the limited attention given to differential effects of demographic characteristics.

## Supplemental Material


Supplemental Material - Tools for the identification of victims of domestic abuse and modern slavery in remote services: A systematic review
Supplemental Material for Tools for the identification of victims of domestic abuse and modern slavery in remote services: A systematic review by Bella Tomsett, Johanna Álvarez-Rodríguez, Nigel Sherriff, Natalie Edelman and Anne Gatuguta in Journal of Health Services Research & Policy.
