# Supplementary material for: Tools for the identification of victims of domestic abuse and modern slavery in remote services: A systematic review
Source: J Health Serv Res Policy. 2024 Jun 7;30(1):63–76. doi: 10.1177/13558196241257864 (PMC11673303; doi:10.1177/13558196241257864)
Supplement: Supplemental Material - Tools for the identification of victims of domestic abuse and modern slavery in remote services: A systematic review [file sj-pdf-1-hsr-10.1177_13558196241257864.pdf]

Journal of Health Services Research & Policy
Tools for the identification of victims of domestic abuse and modern slavery in remote services: A systematic review
Corresponding Author: Tomsett B

Contents

Table S1. Expanded PICO key concepts 1
Table S2. Details of reviewed studies 2
Table S3.Quality assessment of reviewed studies 11

Table S1. Expanded PICO key concepts

Key concepts	Search terms
**KC1 – Domestic Abuse**	“domestic abuse” OR “domestic violence” OR “sexual abuse” OR “sexual violence” OR “partner abuse” OR “spouse abuse” OR “familial abuse” OR “battered women” OR “battered men” OR “intimate partner violence” OR “gender based violence” OR “sexual coercion” OR “relationship coercion” OR “emotional abuse” OR “economic abuse” OR “financial abuse” OR “physical abuse” OR “physical violence” OR “psychological abuse” OR “honour based violence” OR “intimate terrorism”
**KC2 – Modern Slavery**	“modern slavery” OR slavery OR trafficking OR “human trafficking” OR “human smuggling” OR servitude OR “domestic servitude” OR “indentured servitude” OR “bonded labour” OR “sexual exploitation” OR “forced marriage” OR “forced labour” OR “forced work”
**KC3 – Remote services**	telemedicine OR telehealth OR “digital health” OR telecare OR “electronic health” OR “ehealth” OR “mobile health” OR “mhealth” OR helpline OR “telephone consult*” OR “teleconsult*” OR “remote consult*” OR “online consultation” OR “virtual consult*” OR econsult* OR “online clinic” OR “virtual clinic” OR teletriage OR “telephone triage” OR “video conferenc*” OR teleconferenc*
**KC4 – Identification**	Identif* OR assess* OR screen* OR tool OR “risk assess*” OR recogni* OR spot* OR sign* OR pathway* OR safeguard* OR checklist* OR “routine enquiry” OR disclos* OR detect*

Table S2. Details of reviewed studies

No	First author/ Publication date	Study location	Study design	Research aim(s)/ question(s)	Study setting[1]	Abuse type[2]	Participants	Type of tool[3]	Tool purpose[4]	Mode of remote application
1	Fraga, S. **(2014)**	Portugal	Randomised trial	Which mode of delivery is best when screening for IPV in postpartum women, face-to-face, postal, or telephone?	Obstetrics department at an urban hospital.	IPV	829 women who gave birth a year prior.	Questionnaire based on AAS. The questionnaire also collected demographic information, behavioural characteristics and health service utilisation data.	S	Post; phone
2	Tabaie, A. **(2022)**	USA	Observational cohort study	Could a natural language processing (NLP) algorithm be developed that accurately identifies IPV-related encounters from EPR?	Level 1 Trauma Centre in ED.	IPV	405,303 patients visiting ED.	NLP algorithm.	PS	Algorithm
3	Bhargava, R. **(2011)**	USA	Case-control study	To develop a predictive model that identifies clinical profiles of women at high risk of IPV.	Health care services.	IPV	7655 women aged 18-44 registered with the health care provider during the study period.	Predictive model.	PS	Algorithm

4	Gottlieb, L. **(2014)**	USA	Randomised trial	Is there a difference in disclosure rates of psychosocial and socioeconomic adversity between face-to-face and electronic screening?	ED of an urban children's hospital	DV	538 adult primary caregivers seeking treatment for a child at the lower acuity section of the ED.	iScreen - 23 item questionnaire collecting information about psychosocial and socioeconomic problems, adapted from various validated tools, including MASQ from which the questions about domestic violence were adapted.	S	Tablet
5	MacMillan, H. **(2006)**	Canada	Randomised trial	What is the optimal method for IPV screening in health care settings?	ED, family practice, women's health clinic	IPV	2461 women aged 18-64 visiting the health service regarding their own health care.	Questionnaire including the PVS and WAST. In addition, the CAS was used as a criterion standard. Demographic information was also collected.	S	Tablet; written
6	Choo, E. **(2015)**	USA	Qualitative	What are women's attitudes about use of computers for screening and intervening in drug use and partner violence?	ED in urban hospital.	IPV	17 women aged 18-50.	Survey on a broad range of health related topic such as nutrition, access to health care, partner abuse and substance use. WAST was used to screen for partner violence.	S	Tablet
7	Rhodes, K.V. **(2002)**	USA	Descriptive secondary data analysis	Is screening for IPV during computer-based health-risk assessment acceptable to patients, and does it improve detection?	ED in urban hospital	IPV	248 adults aged 18-65.	Survey assessing health risks. 5 IPV screening questions were developed from those previously validated in the AAS and the PVS and from those suggested in the Family Violence	S, INF	Touch-screen computer

								Prevention Fund's Resource Manual for Health Care Providers.		
8	Klevens, J. **(2012)**	USA	Randomised trial	Explore the utility of different strategies used to screen women for IPV and refer those reporting exposure.	Women's health clinics (obstetrical, gynaecological, and family planning clinics) at an urban hospital.	IPV	126 women aged 18 or over.	A-CASI using the PVS tool.	S, INF	Touch-screen computer with headset
9	Ahmad, F. **(2009)**	Canada	Randomised trial	Can computer-assisted screening improve the detection of women at risk for IPV in a family practice setting?	Family practice in urban setting.	IPV	293 adult women in a current or recent intimate relationship. 11 doctors.	Computer programme that administered a previously validated survey of health risks. IPV questions were based on the AAS, PVS and items from Improving the Health Care Response to Domestic Violence: A Resource Manual For Health Care Providers.	S, C, INF	Touch-screen computer
10	O'Campo, P. **(2021)**	Canada	User-centred design study	Develop a suite of mHealth and eHealth apps to facilitate screening for unsafe relationship behaviours and facilitate safety planning to reduce harm of violence.	N/A	IPV	75 women aged 16 and over - 18 for cognitive interviewing: 18; 16 for anonymous encounter surveys; 41 for app user testing.	WithWomen Screener app. HITS instrument was used as the base for the app, with added questions on coercive control and sexual violence.	S, E, INF	App (computer/ mobile)

11	Spence, E.E. **(2022)**	USA	Implementation study	To estimate the proportion of patients who screen positive for IPV and other health indicators	Primary care clinics.	IPV	1495 adult participants.	TESSA mobile web app. IPV screening items include the HITS tool measuring current intimate partner violence, modified questions from the DA instrument that measures high-risk indicators, and other items assessing a history of child abuse, sexual assault, stalking, and past intimate partner violence.	S, INF	Tablet
12	Abujarad, F. **(2021)**	USA	Mixed methods cross-sectional study	To develop and evaluate the usability of a self-administrated digital health tool that screens, educates, and motivates older adults to self-report elder mistreatment.	N/A	EM	38 adults aged 60 and over - 24 for focus groups; 14 for usability testing. 2 clinicans, 3 social workers and 2 caregivers took part in one of the focus groups.	VOICES, a self-administered digital health tool, adapting the EASI-sa screening tool.	S, E, M	Tablet

13	Fincher, D. **(2015)**	USA	Randomised trial	Explore the effect of race-matched vs non-race-matched interviews, and computer-assisted self-interview vs face-to-face interview on disclosure of IPV among African American women.	Women, Infants, and Children (WIC) clinics in urban setting.	IPV	704 women aged 18 and over who are eligible to receive WIC services.	Survey including the revised CTS2S for IPV screening.	S, INF	Computer
14	Scribano, P.V. **(2011)**	USA	Prospective study	Evaluate the feasibility of caregiver-initiated computerised screening in a paediatric ED to identify home safety risks, with a specific emphasis on IPV.	Paediatric ED in urban setting.	IPV	13,057 adult caregivers aged 18-81 accompanying a child to the ED.	Home safety questionnaire. IPV questions included the PVS, screening for physical violence. In addition, 1 item each reflecting emotional and sexual violence was added.	S, INF	Touch-screen kiosk
15	Dado, D. **(2012)**	USA	Mixed-methods comparative study	To compare IPV disclosure in in-person versus computerised screening) among pregnant women in a hospital-based prenatal clinic, and explore the perspectives of the women who had experienced IPV regarding each type of screening	Obstetrics and gynaecology clinics in an urban hospital.	IPV	250 pregnant women aged 18 or over attending their first obstetric appointment.	Questionnaire screening for a variety of behavioural risks including IPV. 6 IPV-related questions were included, screening for physical, emotional and sexual violence, both current and historic.	S	Touch-screen notebook

				method.						
16	Bacchus, L.J. **(2016)**	USA	Nested qualitative interpretative study	Explore perinatal home visitors' and women's experiences of screening for IPV and receiving DOVE via mHealth technology vs a home visitor-led method, and understand whether their perceptions of the technology resulted in differences in the outcomes of its use.	Perinatal home visiting service in urban and rural locations.	IPV	26 women age 16 and over. 23 home visitors and 2 computer programme designers were also interviewed.	DOVE Home Visitation Program. IPV screens used the AAS and WEB scale.	S, E, INF	Tablet
17	Trautman, D.E. **(2007)**	USA	Quasi-experimental study	Would computer-based screening approach result in higher screening, detection, referral, and services for IPV compared to usual care for women who present to the ED?	ED in urban hospital.	IPV	1,005 women aged 18 and over, presenting to ED.	Health survey. IPV questions included PVS to capture physical violence and personal safety, and an addition question was included explicitly asking about sexual abuse.	S, R	Laptop

18	Rhodes, K.V. **(2006)**	USA	Randomised controlled trial	To assess rates of computer-based DV disclosure and to determine the effect of computer screening on DV communication between patients and health care providers during the course of the ED visit.	1 urban and 1 suburban ED.	DV	1281 women aged 18-65. 80 health care providers (30 attending physicians, 46 residents, 4 nurse practitioners)	Promote Health Survey - health risk assessment tool that includes questions about DV based on the AAS and PVS, and modified after cognitive interviewing and field testing.	S, C	Computer
19	Krishnamurti, T. **(2021)**	USA	Observational cohort study	To compare the IPV screening frequencies and IPV incidence rates of patients who started using the app before the COVID-19 shelter-in-place order, to those of patients who started using the app during the shelter-in-place order.	Prenatal care.	IPV	959 pregnant individuals using the mobile app.	MyHealthyPregnancy, an app that predicts likelihood of adverse pregnancy events and assesses psychosocial risk, including IPV. IPV screen included two questions from the Centers for Disease Control and Prevention Behavioral Risk Factor Surveillance System as measures of physical violence and forced sexual acts, and 10 questions from the WEB scale to quantify psychological abuse.	S, INF	Mobile app

20	Gilbert, L. **(2015)**	USA	Randomised controlled trial	To assess the feasibility, safety and efficacy of a single-session computerised self-paced IPV screening, brief intervention and referral to treatment service (SBIRT) in identifying IPV victimisation among women under community supervision and increasing access to IPV services.	Probation services in urban setting.	IPV	191 women aged 18 or over.	WINGS, an A-CASI programme for screening, brief intervention and referral to treatment services. Identification of IPV victimisation was assessed using a shortened 8-item version of the CTS2, measuring any sexual, physical and severe verbal abuse. Severe psychological abuse was assessed with a shortened 8-item version of the PMWI.	S, E, INF, INT	Computer
21	Jack, S.M. **(2021)**	Canada/USA/UK	Policy analysis – based design study	Explore how current, global evidence-informed guidelines that support nurses and midwives to recognise and respond to IPV can be translated from face-to-face encounters to care via telehealth.	N/A	IPV	N/A	Guidance with suggested scripts.	S	Remote consultation
22	Simon, M.A. **(2021)**	USA	N/A	Explore how to identify and support people experiencing IPV during telehealth encounters.	Health care services.	IPV	N/A	Guidance with suggested scripts.	S, E, INF	Remote consultation

23	Bair-Merritt, M.H. **(2006)**	USA	Randomised trial	To compare women's acceptability ratings of 2 different IPV screening methods, an audiotape questionnaire and a written questionnaire, in a paediatric emergency department.	ED in urban children's hospital.	IPV	499 women caregivers aged 18 or over attending the ED with a child.	Safety screening questionnaire comprised of 10 questions. The 4 IPV questions were based on the FPVS, and an additional question assessing emotional abuse from an IPV instrument validated in an ED population of low-income African American women.	S	Audiotape and headset
24	Ragavan, M.I. **(2020)**	USA	N/A	To offer recommendations to support IPV survivors and their children during the current pandemic and in the foreseeable future, as more health care is delivered virtually.	Paediatric health care.	IPV	N/A	Guidance with suggested scripts.	S, E, INF	Remote consultation

Abbreviations:
[1] **ED** - Emergency Department
[2] **IPV** - Intimate Partner Violence; **DV** - Domestic Violence; **EM** - Elder Mistreatment

[3] **AAS** - Abuse Assessment Screen; **MASQ** - Medical Advocacy Screening Questionnaire; **PVS** - Partner Violence Screen; **WAST** - Woman Abuse Screening Tool; **CAS** - Composite Abuse Scale;
A-CASI - Audio Computer-Assisted Self-Interviews; **HITS** - Hurt, Insult, Threaten, and Scream; **DA** - Danger Assessment; **EASI-sa** - Elder Abuse Suspicion Index; **CTS2S** - Conflict Tactics Scales Short Form; **WEB** - Women's Experience with Battering; **CTS2** - Revised Conflict Tactics Scale; **PMWI** - Psychological Maltreatment against Women Inventory; **FPVS** - Feldhaus Partner Violence Screen
[4] **S** - screening; **INF** - information; **E** - education; **PS** - pre-screening; **M** - motivation; **C** - conversation; **INT** - intervention; **R** - referral

Journal of Health Services Research & Policy
Tools for the identification of victims of domestic abuse and modern slavery in remote services: A systematic review
Corresponding Author: Tomsett B

Table S3.Quality assessment of reviewed studies

QuADS criteria	Q1	Q2	Q3	Q4	Q5	Q6	Q7	Q8	Q9	Q10	Q11	Q12	Q13	Total
**Study**														**(out of 39)**
Abujarad, F., et al. (2021)	2	2	1	3	1	1	3	3	1	0	0	1	1	19
Ahmad, F., et al. (2009)	1	3	3	3	3	3	3	3	3	3	3	2	3	36
Bacchus, L.J., et al. (2016)	2	3	3	3	3	3	2	3	2	1	3	0	2	30
Bair-Merritt, M.H., et al. (2006)	0	3	3	2	1	3	3	2	2	3	2	2	1	27
Bhargava, R., et al. (2011)	0	2	3	3	2	0	0	2	3	0	3	0	1	19
Chang, J., et al. (2012)	0	2	2	3	1	0	3	2	1	0	2	1	2	19
Choo, E., et al. (2015)	1	3	3	3	2	3	2	1	1	0	3	0	2	24
Fincher, D., et al. (2015)	1	3	3	3	3	2	3	3	2	3	3	0	3	32
Fraga, S., et al. (2014)	0	2	3	2	1	1	2	1	3	0	1	0	1	17
Gilbert, L., et al. (2015)	0	3	3	2	1	0	3	2	3	2	3	2	1	25
Gottlieb, L., et al. (2014)	0	3	3	3	1	2	3	3	3	0	3	0	3	27
Jack, S.M., et al. (2021)	3	3	2	2	3	3	3	2	3	0	3	1	1	29
Klevens, J., et al. (2012)	1	3	3	2	1	3	2	3	3	0	2	0	3	26
Krishnamurti, T., et al. (2021)	0	2	3	2	1	1	2	1	3	0	2	0	2	19
MacMillan, H. L., et al. (2006)	0	3	3	3	3	3	2	3	3	2	3	0	3	31
O'Campo, P., et al. (2021)	2	3	2	2	1	1	2	2	1	0	2	3	1	22
Rhodes, K.V., et al. (2002)	1	3	3	2	1	2	2	1	3	0	1	1	2	22
Rhodes, K.V., et al. (2006)	0	3	3	3	1	2	3	2	3	0	3	0	1	24
Scribano, P.V., et al. (2011)	1	3	2	2	1	2	2	2	2	2	2	2	2	25
Spence, E.E., et al. (2022)	1	2	3	2	0	1	3	1	1	0	0	0	0	14
Tabaie, A., et al. (2022)	1	2	2	3	2	3	3	3	0	0	3	1	3	26
Trautman, D.E., et al. (2007)	1	3	3	2	3	3	3	3	2	2	2	0	3	30

***Scoring: 0-3; 0 = criterion not met at all, 3 = criterion met fully**
